# Exploratory Analysis of Autophagy–Lysosomal Pathway Proteins in Dermal Fibroblasts as Potential Peripheral Biomarkers for Alzheimer’s Disease: A Pilot Study

**DOI:** 10.3390/biomedicines14010034

**Published:** 2025-12-23

**Authors:** Myung Shin Lee, Sang Joon Son, Juyeong Kim, Seungbeom Go, Chang Hyung Hong, Hyun Woong Roh, Jaerak Chang

**Affiliations:** 1 Department of Biomedical Sciences, Ajou University School of Medicine, Suwon 16499, Republic of Korea; myeongshin87@ajou.ac.kr (M.S.L.); juyeongk@ajou.ac.kr (J.K.); rhtmdqja@ajou.ac.kr (S.G.); 2 Department of Psychiatry, Ajou University School of Medicine, Suwon 16499, Republic of Korea; sjsonpsy@ajou.ac.kr (S.J.S.); antiaging@ajou.ac.kr (C.H.H.); hansin8607@naver.com (H.W.R.); 3 Department of Brain Science, Ajou University School of Medicine, Suwon 16499, Republic of Korea

**Keywords:** Alzheimer’s disease, autophagy-lysosomal pathway, biomarkers, dermal fibroblasts, early diagnosis

## Abstract

**Background/Objectives:** Alzheimer’s disease (AD) is characterized by accumulation of abnormal intracellular substances and autophagy–lysosomal pathway (ALP) dysfunction. While current diagnostic methods rely on cerebrospinal fluid biomarkers and neuroimaging, minimally invasive peripheral biomarkers are needed. Dermal fibroblasts could serve as accessible reporters of AD-related molecular changes. This exploratory pilot study investigated whether ALP-associated proteins in patient-derived fibroblasts could serve as potential peripheral biomarkers for AD diagnosis. **Methods:** We analyzed dermal fibroblasts from 9 AD patients (amyloid Positron emission tomography (PET)-positive) and 9 age-matched controls (amyloid PET-negative). Comprehensive immunoblot analysis assessed expression profiles of 16 AD- and ALP-associated proteins. Autophagic flux and lysosomal function were evaluated using bafilomycin A1 treatment and LysoTracker staining. Diagnostic performance was assessed through receiver operating characteristic (ROC) curve analysis and multivariable logistic regression. **Results:** AD fibroblasts showed significantly reduced Beta-site APP cleaving enzyme 1 (BACE1) (*p* = 0.022) and elevated Tax1-binding protein 1 (TAX1BP1) (*p* = 0.035) expression. BCL2-associated athanogene proteins 2 (BAG2) and OPTN demonstrated consistent directional changes across patients. Preliminary ROC analysis showed promising performance for protein combinations, with BAG2 + OPTN achieving Area under the curve (AUC) = 0.963 (sensitivity 77.8%, specificity 88.9%). Integration with Apolipoprotein E4 (*APOE4*) status further enhanced diagnostic accuracy (*APOE4* + BACE1: AUC = 0.914). Notably, baseline autophagic flux and lysosomal acidification were preserved, suggesting pathway-specific rather than systemic ALP dysfunction. **Conclusions:** This exploratory study provides preliminary evidence that dermal fibroblast-derived ALP proteins show disease-associated alterations in AD and may represent potential peripheral biomarkers. However, given the small sample size (*n* = 18) and lack of independent validation, these findings require confirmation in larger multi-center cohorts before clinical translation.

## 1. Introduction

Alzheimer’s disease (AD) is the most prevalent neurodegenerative disorder worldwide, characterized by progressive cognitive decline and the accumulation of two major pathological hallmarks: extracellular amyloid-beta (Aβ) plaques and intracellular neurofibrillary tangles composed of hyperphosphorylated tau protein [1,2]. While most cases occur after age 65 (late-onset AD, LOAD), a small percentage develop symptoms earlier (early-onset AD, EOAD), often due to genetic mutations in Amyloid precursor protein (*APP*), Presenilin 1 (*PSEN1*), or Presenilin 2 (*PSEN2*) [3]. The complex pathophysiology of AD involves multiple cellular processes, including protein aggregation, mitochondrial dysfunction, oxidative stress, and neuroinflammation, making early diagnosis and development of treatment particularly challenging.

Current diagnostic methods for AD rely heavily on cerebrospinal fluid (CSF) biomarkers and neuroimaging techniques [4]. Although these approaches offer high diagnostic accuracy, they are invasive, expensive, and often detect the disease only after significant neurodegeneration has occurred [5]. Recent advances in highly sensitive immunoassay platforms (e.g., Single molecule array (SIMOA)) and mass spectrometry have led to remarkable progress in blood-based AD biomarkers, with several studies demonstrating that blood Aβ and phosphorylated tau (p-tau) species exhibit robust diagnostic performance [6]. Plasma p-tau217 and p-tau181 have shown particularly promising results in distinguishing AD from other neurodegenerative disorders, highlighting the potential of peripheral biomarkers in AD diagnosis [6,7]. Given this emerging importance of peripheral markers, there is growing interest in identifying additional minimally invasive biomarkers that could complement existing blood-based tests and provide deeper insights into AD pathophysiology. In this context, cellular biomarkers that reflect disease-specific molecular alterations could offer unique opportunities for both mechanistic understanding and diagnostic applications.

The autophagy–lysosomal pathway (ALP) plays a fundamental role in protein quality control and cellular homeostasis [8]. This system is responsible for the degradation of damaged organelles, protein aggregates, and other cellular components, making it crucial for maintaining neuronal health. Mounting evidence suggests that ALP dysfunction is an early and crucial event in AD pathogenesis [9,10]. Impaired autophagy leads to the accumulation of toxic protein aggregates, particularly Aβ and tau, contributing to neurodegeneration [11]. Recent studies have shown that alterations in ALP-related proteins, such as cathepsin D, may serve as potential biomarkers for AD, as they show distinct changes in peripheral fluids [12].

Importantly, these pathological changes are not confined to the brain but can be detected in peripheral tissues, suggesting AD is a systemic disease [13,14]. This systemic nature of AD pathology opens new possibilities for developing peripheral biomarkers that could reflect disease status and progression. Understanding the relationship between central and peripheral manifestations of AD could provide valuable insights into disease mechanisms and potential therapeutic targets.

Dermal fibroblasts have emerged as a promising tool for studying neurodegenerative diseases, as they share many cellular pathways with neurons and can be obtained through minimally invasive procedures [15]. Several studies have demonstrated that fibroblasts from AD patients exhibit various cellular abnormalities, including altered calcium homeostasis, mitochondrial dysfunction, and enhanced oxidative stress [16,17]. The preservation of disease-specific phenotypes in these cells makes them excellent candidates for biomarker discovery and mechanistic studies.

Recent evidence indicates that AD patients’ fibroblasts show distinct alterations in protein degradation pathways, suggesting they might serve as peripheral reporters of disease status [18]. Furthermore, these cells maintain their phenotypic characteristics in culture, allowing for detailed mechanistic studies of disease-related pathways [19]. The accessibility and stability of fibroblasts, combined with their ability to reflect disease-specific alterations, make them attractive candidates for biomarker development. The analysis of ALP components in these cells could provide valuable insights into AD pathogenesis and potentially lead to the development of novel diagnostic tools.

Despite major advances in blood-based biomarkers, there remains a need for complementary, mechanistically informative peripheral readouts that capture cell-intrinsic disturbances in proteostasis pathways relevant to AD. In this exploratory pilot study, we investigated the potential of AD- and ALP-associated proteins in patient-derived fibroblasts as peripheral biomarkers for AD. Using dermal fibroblasts from 9 AD patients (amyloid Positron emission tomography (PET)-positive) and 9 age-matched controls (amyloid PET-negative), we performed comprehensive proteomic analyses examining the expression profiles of key proteins involved in AD pathology and autophagy regulation. We also assessed autophagic function through cellular assays to evaluate pathway-specific alterations. Our findings reveal distinct molecular signatures in AD patient fibroblasts, suggesting these cells may serve as accessible peripheral reporters of disease-associated changes. While this preliminary investigation has limitations inherent to small sample size, the identified protein alterations and their diagnostic potential warrant validation in larger independent cohorts. The development of reliable peripheral biomarkers could complement existing diagnostic approaches and facilitate earlier AD detection, particularly in resource-limited settings where advanced neuroimaging or CSF collection may not be readily available. By establishing feasibility of multi-marker profiling and functional ALP readouts in subject-derived fibroblasts, this study provides a practical framework for future validation and for integrating cellular signatures with established fluid biomarkers.

## 2. Materials and Methods

### 2.1. Subjects and Study Approval

This study was a part of the ongoing Biobank Innovations for chronic Cerebrovascular disease With Alzheimer’s disease Study (BICWALZS) and the Center for Convergence Research on Neurological Disorders [20]. Dermal fibroblast samples were obtained from the Biobank Center at Ajou University Hospital. The study cohort consisted of 9 AD patients (amyloid PET-positive) and 9 age-matched controls with non-demented mild cognitive impairment (amyloid PET-negative) [21,22]. Amyloid PET positivity was determined quantitatively using 18F-flutemetamol PET scans. Standard uptake value ratios (SUVRs) were calculated using the pons as a reference region. Global cortical 18F-flutemetamol retention was computed as the volume-weighted average SUVR from bilateral cortical regions including frontal, posterior cingulate, lateral temporal, parietal, and occipital lobes, based on the annotated anatomical labeling atlas. Participants were classified as amyloid-positive when their global cortical SUVR exceeded 0.634, a threshold established based on previous studies in the elderly Korean population [20]. To minimize potential confounding effects from vascular pathology, only subjects with White Matter Hyperintensities (WMH) scores 1 were included. WMH scores were rated visually by two psychiatrists and six neurologists. Subjects with significant psychiatric illness, other neurodegenerative disorders, or severe systemic diseases were excluded. The study protocol was approved by the Institutional Review Board of Ajou University Hospital (AJOUIRB-SUR-2021-038), and written informed consent was obtained from all participants or their legal representatives. All participants were Koreans of Eastern Asian ethnicity and were recruited from the Ajou University Hospital.

### 2.2. APOE Genotyping

Genomic DNA was isolated from blood and single-nucleotide polymorphism (SNP) genotyping was conducted by DNA Link, Inc. using the Affymetrix Axiom KKORV1.0-96 Array (Thermo Fisher Scientific, Waltham, MA, USA) according to manufacturer’s protocol. Apolipoprotein E4 (*APOE4*) was derived from rs429358 and rs7412.

### 2.3. Antibodies

The following primary antibodies were used: alpha-Tubulin (Abcam, Cambridge, UK; ab18251), APP-CTF (Abcam, Cambridge, UK; ab32136), Beta-site APP cleaving enzyme 1 (BACE1) (ProteinTech Group, Rosemont, IL, USA; 12807-1-AP), BCL2-associated athanogene proteins 2 (BAG2) (Bethyl, Montgomery, TX, USA; A304-751A), BCL2-associated athanogene proteins 3 (BAG3) (ProteinTech Group, Rosemont, IL, USA; 10599-1-AP), Cathepsin D (Calbiochem, San Diego, CA, USA; IM-03), Early endosome antigen 1 (EEA1) (BD Biosciences, San Jose, CA, USA; 610456), Glucose-regulated protein 78 (GRP78) (BD Biosciences, San Jose, CA, USA; 610978), Lysosome-associated membrane protein 2A (LAMP2A) (Abcam, Cambridge, UK; ab18528), Microtubule-associated protein 1 light chain 3 (LC3) (Sigma, St. Louis, MO, USA; L8918), Neighbor of BRCA1 gene 1 (NBR1) (ProteinTech Group, Rosemont, IL, USA; 16004-1-AP), Optineurin (OPTN) (Bethyl, Montgomery, TX, USA; A301-831A), PSEN2 (Bethyl, Montgomery, TX, USA; A304-342A), RAS-Associated Protein RAB7A (RAB7) (Cell Signaling Technologies, Danvers, MA, USA; 9367), Tax1-binding protein 1 (TAX1BP1) (ProteinTech Group, Rosemont, IL, USA; 14424-1-AP), Transcription factor E3 (TFE3) (Bethyl, Montgomery, TX, USA; A302-622), Toll-interacting protein (TOLLIP) (ProteinTech Group, Rosemont, IL, USA; 11315-1-AP), Ubiquitin (ProteinTech Group, Rosemont, IL, USA; 10201-2-AP).

### 2.4. Dermal Fibroblast Isolation and Culture

Dermal fibroblasts were obtained from skin biopsies collected within four weeks of baseline assessment from consenting participants. Following established protocols [23], a 3 mm^3^ punch biopsy was performed on the upper inside arm under local anesthesia using aseptic techniques. The tissue was immediately transferred to pre-warmed culture medium and processed in the laboratory. Biopsy specimens were mechanically dissected into small pieces in a 100 mm culture dish with minimal medium to facilitate tissue attachment. After brief attachment (1 min), Dulbecco’s Modified Eagle Medium (DMEM; GenDEPOT, Baker, TX, USA, CM001-050) supplemented with 20% fetal bovine serum (FBS; GenDEPOT, Baker, TX, USA, F0900-050) and 1% penicillin/streptomycin was added. Culture medium was changed every 2–3 days after the first week. Upon sufficient fibroblast outgrowth from tissue fragments, cells were trypsinized and subcultured (passage 1). Primary dermal fibroblasts were maintained at 37 °C in a humidified atmosphere containing 5% CO_2_. Cells between passages 3–8 were used for all experiments to minimize potential effects of cellular senescence. Population doubling levels were monitored throughout the culture period.

### 2.5. Immunoblotting

Immunoblot analysis was performed as follows, based on our previously described protocols [24,25]. Fibroblasts were washed once with PBS and lysed in 1× SDS buffer (50 mM Tris-HCl pH 7.4, 2% SDS and 10% glycerol). Cell lysates were heated at 95 °C for 10 min. Equal amounts of protein were resolved by sodium dodecyl sulfate-polyacrylamide gel electrophoresis (SDS-PAGE) and transferred onto nitrocellulose membranes (GE Healthcare Life Science, Marlborough, MA, USA; 10600001). All samples were processed using the same protocol and reagents. Due to gel capacity and limited sample availability, some targets were assessed on separate gels. Equal sample loading was verified by Ponceau S (Biosesang, Yongin, Republic of Korea; P1031) staining. Membranes were blocked in 3% non-fat milk in Tris-buffered saline (TBS; 20 mM Tris-HCl, 137.5 mM NaCl, pH 7.6) with 0.05% Tween-20 (TBST) for 30 min and incubated with primary antibodies in TBST with 3% bovine serum albumin (BSA) at 4 °C overnight. Blots were washed three times with TBST, incubated with horseradish peroxidase-conjugated secondary antibodies (SouthernBiotech, Birmingham, AL, USA; 1031-05 and 4055-05) for 1 h, and washed three times with TBST. Clarity Western ECL Substrate (Bio-Rad Laboratories, Hercules, CA, USA) was used to detect immunoreactive proteins. Images were obtained and processed using a ChemiDoc XRS + System with Image Lab software v5.2 (Bio-Rad Laboratories, Hercules, CA, USA). Each gel included samples from both groups, and a common reference sample was included across gels to reduce between-gel variation.

### 2.6. Densitometric Analysis and Protein Quantification

**Figure 1:**
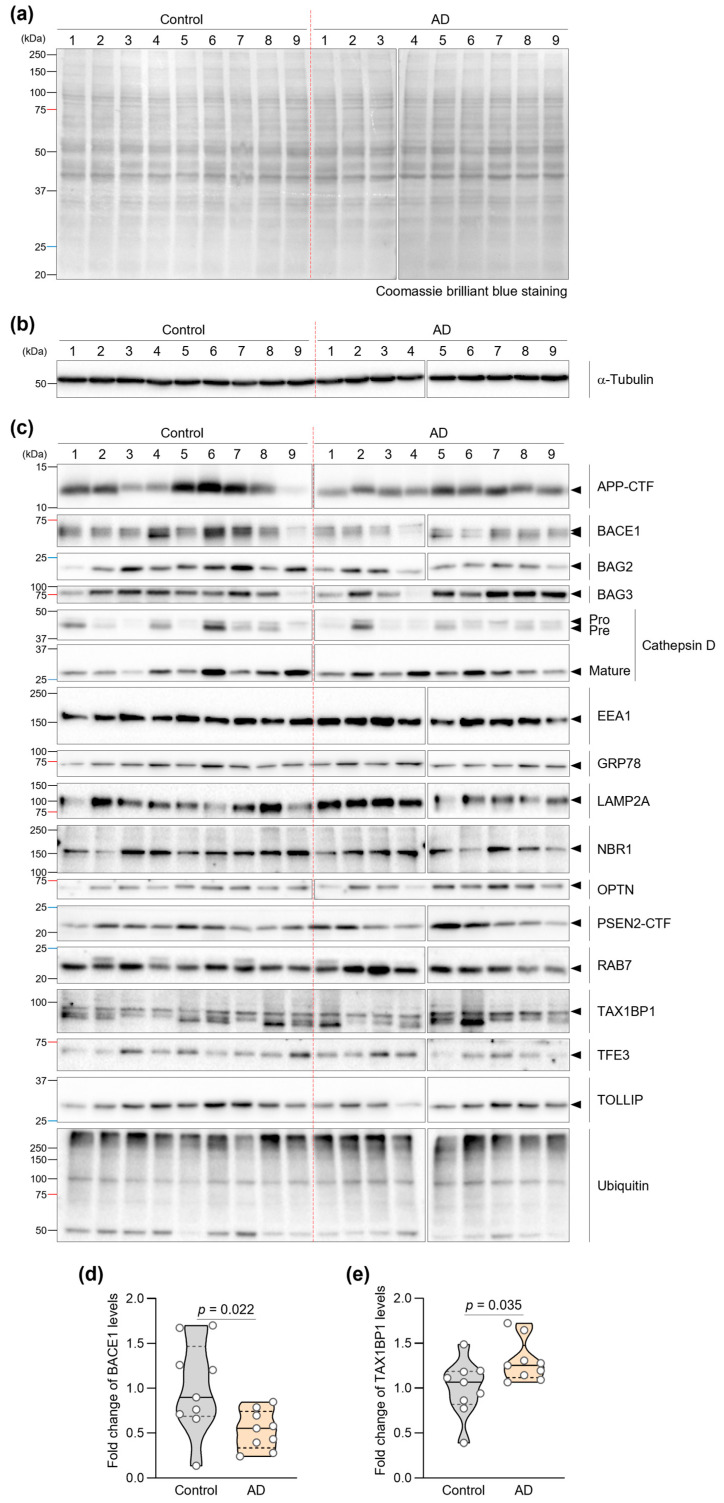
Expression profiles of AD- and ALP-associated proteins in AD patient fibroblasts. (**a**) Representative images of Coomassie brilliant blue-stained membranes showing equal protein loading across samples from control and AD patients. (**b**) Representative immunoblot images of α-Tubulin used as a loading control to verify equal protein loading in fibroblasts from control and AD patients. (**c**) Immunoblot analysis of AD- and ALP-associated proteins (APP, BACE1, BAG2, BAG3, Cathepsin D, EEA1, GRP78, LAMP2A, NBR1, OPTN, PSEN2, RAB7, TAX1BP1, TFE3, TOLLIP, and Ubiquitin) in fibroblasts from control (*n* = 9) and AD patients (*n* = 9). (**d**,**e**) Quantification of BACE1 (**d**) and TAX1BP1 (**e**) protein levels. Statistical significance was determined using Mann–Whitney U test (*p* < 0.05). Violin plots show the distribution; overlaid dots represent individual subject-derived fibroblast lines (biological replicates). The central line indicates the median and the bounds of the box indicate the interquartile range (IQR).

Protein expression levels were quantified using densitometric analysis. Equal protein loading (8 μg per lane) was verified by Coomassie brilliant blue staining of parallel gels. Coomassie Brilliant Blue staining of a parallel gel was used solely for qualitative verification of comparable total protein loading and was not used for quantitative normalization. In addition, α-Tubulin was assessed as a loading control (Figure 1b) to confirm equivalent loading across lanes. Image Lab software v5.2 (Bio-Rad Laboratories, Hercules, CA, USA) was used for densitometric analysis of immunoblots. To minimize technical variation, control and AD samples were processed in parallel under identical conditions. For final quantification, the normalized densitometric values for each sample were expressed as relative expression levels by dividing by the mean value of the control group (*n* = 9). These relative expression values were used for statistical analyses and graphical presentation, with comparisons performed using the Mann–Whitney U test. Results are presented as median ± 95% confidence interval.

### 2.7. Autophagic Flux Analysis

Autophagic flux was assessed by monitoring LC3-II accumulation in response to lysosomal inhibition. Cells were treated with either DMSO (control) or 400 nM bafilomycin A1 (Sigma, St. Louis, MO, USA; B1793) for 3 h before protein extraction. Autophagy flux was calculated as the ratio of LC3-II levels in bafilomycin A1-treated versus DMSO-treated cells. For LC3-II quantification, band intensities were first normalized to α-Tubulin within each lane, and autophagic flux was calculated as the ratio of normalized LC3-II levels in Baf. A1-treated versus DMSO-treated cells. LC3-II levels were analyzed using a two-factor mixed-effects model (GraphPad Prism 8.0), with treatment (DMSO vs. Baf. A1) as a within-subject repeated factor and group (control vs. AD) as a between-subject factor, and fibroblast ID entered as a random effect. Because the repeated factor had only two levels, sphericity assumptions were not applicable.

### 2.8. Lysosomal pH Assessment

Lysosomal pH was evaluated using LysoTracker™ Red DND-99 (Invitrogen, Carlsbad, CA, USA; L7528). Cells were incubated with 800 nM LysoTracker for 30 min at 37 °C, then harvested by trypsinization and resuspended in PBS. Fluorescence intensity was measured using a BD FACSAria III flow cytometer, and at least 10,000 single-cell events were collected and analyzed per sample. Data analysis was performed using FACSDiva software 8.0.3 (BD Biosciences, San Jose, CA, USA).

### 2.9. Statistical Analysis

Each biological replicate represents one subject-derived fibroblast line; assays were performed once per line (technical replicates = 1). Statistical analyses were performed using GraphPad Prism 8.0, IBM SPSS Statistics v25.0 and R software version 4.01. Normal distribution was assessed using the Shapiro–Wilk test. For demographic and clinical characteristics, categorical variables (sex and *APOE4* carrier status) were analyzed using Fisher’s exact test, while continuous variables (age, education duration, Clinical Dementia Rating (CDR) and CDR Sum of Boxes (CDR-SB)) were compared using Mann–Whitney U test. For protein expression analysis, group comparisons were conducted using the Mann–Whitney U test for non-parametric data. For LC3-II–based autophagic flux experiments, we analyzed normalized LC3-II levels using a two-factor mixed-effects model (GraphPad Prism 8.0), with Treatment (DMSO vs. bafilomycin A1) as a within-subject repeated factor and Group (control vs. AD) as a between-subject factor, including fibroblast ID as a random effect; the Group × Treatment interaction term was used as the primary test of differential flux responses between groups. Results are presented as median ± 95% confidence interval. Correlations between variables were evaluated using Spearman’s rank correlation coefficient. For diagnostic performance assessment, receiver operating characteristic (ROC) curves were generated and area under the curve (AUC) values were calculated. Cutoff values for ROC curve analysis were determined using Youden’s index, which maximizes the sum of sensitivity and specificity. Logistic regression analysis was performed to evaluate combinations of biomarkers. Pearson’s chi-square test was used to evaluate categorical variables. In particular, for multivariable logistic regression models with two predictors, the events-per-variable (EPV) was well below commonly recommended thresholds, which further increases the risk of overfitting and overly optimistic estimates of model performance. Given the exploratory and hypothesis-generating nature of this pilot study, we did not apply correction for multiple comparisons across the 16 proteins analyzed. This decision reflects our aim to identify candidate biomarkers for future validation rather than establish definitive disease markers. Consequently, all reported *p*-values should be interpreted as descriptive statistics to guide hypothesis generation, and findings require confirmation in independent cohorts with appropriate multiple testing corrections. We acknowledge that the limited sample size (*n* = 18 total) constrains statistical power and increases the risk of both type I and type II errors. The diagnostic performance metrics (AUC, sensitivity, specificity) derived from ROC analysis may be subject to optimism bias due to the small sample size and lack of independent validation cohort. These preliminary performance estimates should be interpreted cautiously and require validation through cross-validation methods or independent external datasets in future studies. Statistical significance was set at *p* < 0.05 for descriptive purposes. All statistical tests were two-tailed.

## 3. Results

### 3.1. Demographic and Clinical Characteristics of Subjects

In this exploratory case–control study, we compared 9 patients with AD confirmed by amyloid PET positivity and 9 age-matched controls with negative amyloid PET scans. To minimize the potential confounding effects of cerebrovascular pathology, we selected participants with white matter hyperintensities (WMH) scores ≤ 1. The mean age was comparable between groups (AD: 68.2 ± 5.3 years, Control: 69.8 ± 4.5 years, *p* = 0.395), and both groups showed a similar gender distribution with female predominance (Table 1). Education duration was similar between groups (AD: 6.8 ± 6.4 years, Control: 9.4 ± 2.9 years, *p* = 0.161). Notably, *APOE4* carriers were more prevalent in the AD group (77.8%, 7/9) compared to controls (22.2%, 2/9; *p* = 0.057). As expected, AD patients exhibited significantly higher CDR and CDR-SB scores than controls (*p* < 0.001), confirming their clinical disease status.

### 3.2. Distinct Expression Patterns of AD- and ALP-Associated Proteins in AD Patient Fibroblasts

To investigate potential alterations in the ALP, a key pathogenic mechanism in AD [26], we conducted a comprehensive exploratory analysis of AD- and ALP-associated proteins in patient-derived skin fibroblasts from our small cohort (*n* = 9 per group). The selected proteins represent key components of distinct but interconnected cellular pathways: (1) AD pathology-related proteins: APP, BACE1, PSEN2 [27,28]; (2) Autophagy machinery: BAG2, BAG3, EEA1, LC3, NBR1, OPTN, RAB7, TAX1BP1, TFE3, TOLLIP, Ubiquitin [29,30,31,32,33,34,35,36,37,38,39]; (3) Lysosomal components and regulators: LAMP2A, Cathepsin D, GRP78 [34,37,38].

Equal protein loading was confirmed by Coomassie brilliant blue staining and immunoblot of α-Tubulin (Figure 1a,b). Quantitative immunoblot analysis revealed significant alterations in two proteins (Figure 1c). Most notably, BACE1 levels were reduced by 47.2% in AD fibroblasts compared to controls (*p* = 0.022) (Figure 1d). Additionally, we observed a 28.5% increase in TAX1BP1 levels in AD fibroblasts (*p* = 0.035) (Figure 1e). TAX1BP1, which facilitates autophagic clearance of protein aggregates [36], was increased in AD fibroblasts compared with controls (*p* = 0.035).

Several other proteins demonstrated consistent directional changes across individual AD patient samples, though these did not achieve statistical significance in our cohort. Specifically, OPTN showed a 25% increase (*p* = 0.078) and BAG2 exhibited a 32% decrease (*p* = 0.052) in AD fibroblasts. Notably, examining individual patient data revealed that 8 out of 9 AD patients showed elevated OPTN expression relative to the control group median, and 8 out of 9 AD patients showed reduced BAG2 expression (Supplementary Materials Figure S1). Although our limited sample size (*n* = 18) prevented these differences from reaching statistical significance, consistent directional trends were observed across individual samples, and these preliminary observations require validation in larger independent cohorts. The remaining analyzed proteins showed no significant differences or consistent patterns between groups (Table 2 and Supplementary Materials Figure S1).

### 3.3. Analysis of Autophagic Activities in AD Patient Fibroblasts

**Figure 2:**
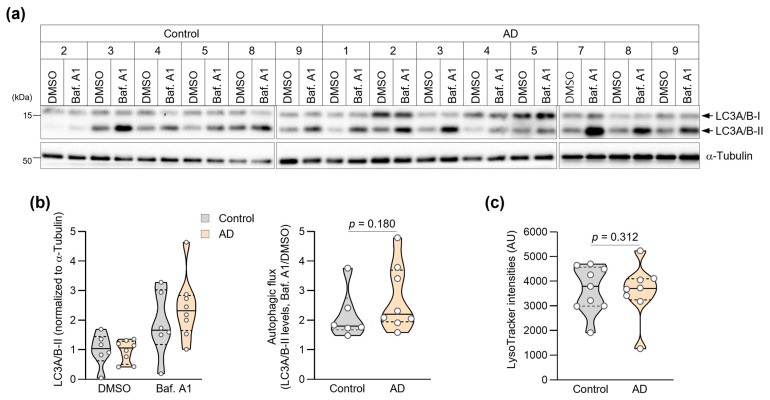
Assessment of autophagic activities in AD patient fibroblasts. (**a**) Immunoblot showing LC3A/B expression in fibroblasts treated with DMSO or bafilomycin A1 (Baf. A1, 400 nM) for 3 h. Molecular weight markers (kDa) are indicated. (**b**) Quantification of autophagic flux measured individual values of LC3-I and LC3-II. Autophagy flux measured the ratio of LC3-II levels in Baf. A1-treated versus DMSO-treated cells (Control: *n* = 6, AD: *n* = 8). (**c**) Quantification of mean LysoTracker fluorescence intensity per cell analyzed by flow cytometry (>10,000 cells per sample; Control: *n* = 9, AD: *n* = 8). Statistical comparisons of autophagic flux were based on the Group × Treatment interaction from a two-factor mixed-effects model (see Methods). Data in (**b**,**c**) are presented as violin plots with overlaid individual values. The *p*-value in (**b**) is derived from the Group × Treatment interaction term in the two-factor mixed-effects model. Violin plots show the distribution; overlaid dots represent individual subject-derived fibroblast lines (biological replicates). The central line indicates the median and the bounds of the box indicate the interquartile range (IQR) with *p*-values determined by Mann–Whitney U test.

Given the crucial role of autophagy in protein quality control and its dysfunction in AD [26], we examined autophagic function in patient fibroblasts using two complementary approaches in this preliminary investigation. First, we assessed autophagic flux by measuring LC3-II accumulation in response to bafilomycin A1, a specific inhibitor of vacuolar H+-ATPase that blocks autophagosome–lysosome fusion. Autophagic flux was assessed only in lines for which paired DMSO and bafilomycin A1 measurements could be obtained. Due to limited patient-derived material/cell yield, three control lines and one AD line could not be assayed under both conditions and were excluded, leaving *n* = 6 (control) and *n* = 8 (AD). Due to limited patient-derived material/cell yield, one AD line could not be included in the LysoTracker assay, resulting in control (*n* = 9) and AD (*n* = 8) for this analysis. The mixed-effects model revealed a significant main effect of Treatment (F(1,12) = 30.33, *p* = 0.0001), indicating robust LC3-II accumulation upon Baf. A1 treatment across groups. The main effect of Group was not significant (F(1,12) = 0.40, *p* = 0.54). Importantly, the Group × Treatment interaction was not significant (F(1,12) = 2.00, *p* = 0.18), suggesting that the magnitude of LC3-II accumulation was comparable between control and AD fibroblasts. Notably, we found no significant difference in autophagic flux between fibroblasts from AD patients and controls (Figure 2a,b), suggesting that the baseline rate of autophagosome formation remains intact in AD patient cells.

We then evaluated lysosomal function by measuring lysosomal acidification using LysoTracker™ Red DND-99, a fluorescent dye that specifically accumulates in acidic organelles. Proper lysosomal acidification is essential for the activity of lysosomal hydrolases and efficient protein degradation [40]. Flow cytometric analysis of over 10,000 cells per sample revealed comparable LysoTracker fluorescence intensities between fibroblasts from AD patients and controls (Figure 2c and Figure S2). Consistently, mean LysoTracker fluorescence intensity did not differ significantly between groups (Control: median 3792, 95% CI 2981–4646, *n* = 9; AD: 3699, 95% CI 1260–5234, *n* = 8; Mann–Whitney U test, *p* = 0.870; Figure 2c and Table 2). At the current sample size, we did not detect statistically significant differences in lysosomal acidification between groups, and these negative findings should be interpreted with caution.

### 3.4. Correlation Analysis of AD- and ALP-Associated Features in AD Patient Fibroblasts

**Figure 3:**
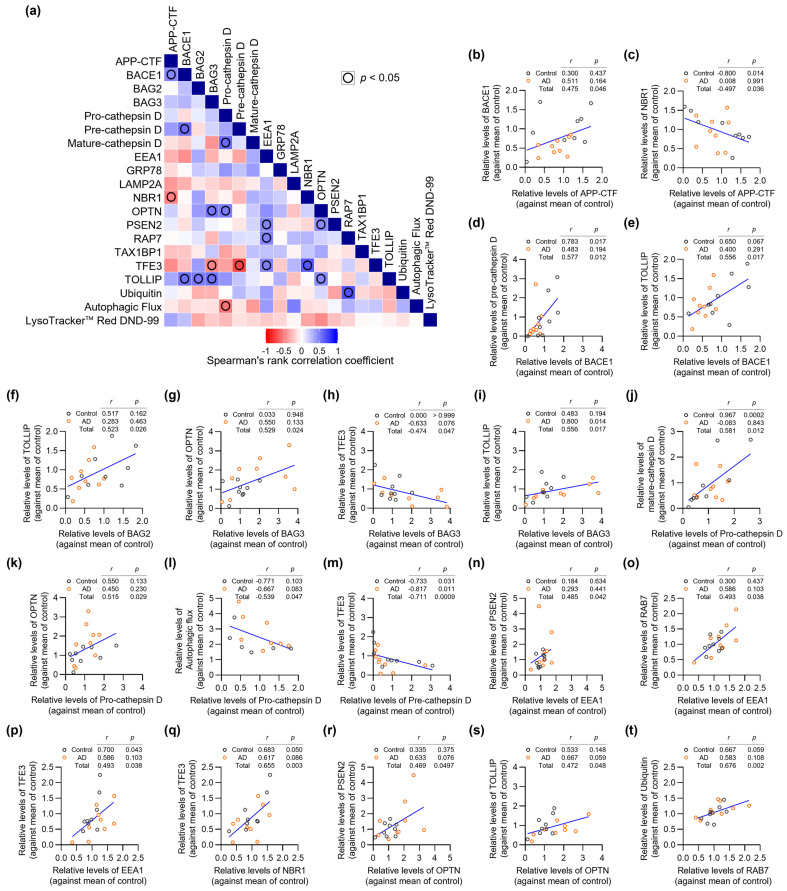
Correlation analysis of AD- and ALP-associated features in AD patient fibroblasts. (**a**) Correlation matrix heatmap showing Spearman’s rank correlation coefficients between all analyzed parameters from the exploratory cohort (*n* = 18). Blue indicates positive correlations, red indicates negative correlations. (**b**–**t**) Scatter plots showing significant correlations between parameters. Each point represents an individual patient sample (Control: *n* = 9, AD: *n* = 9). Spearman’s rank correlation coefficients (*r*) and *p*-values (*p*) are shown for each correlation pair. Given the small sample size, some correlations may be influenced by individual extreme observations and should be interpreted as exploratory.

To gain insights into the potential interplay between AD- and ALP-associated proteins, we performed exploratory correlation analysis of protein expression levels across all samples (*n* = 18) (Figure 3 and Supplementary Materials Table S1). Particularly notable was the significant positive correlation between APP-CTF and BACE1 levels (Figure 3b), which aligns with the established role of BACE1 in APP processing [28]. We observed an unexpected negative correlation between APP-CTF and NBR1 (Figure 3c). While NBR1 is known to participate in selective autophagy, this finding suggests it may also play a previously unrecognized role in APP-CTF clearance.

BAG3 emerged as a central player, showing strong positive correlations with multiple autophagy regulators including OPTN and TOLLIP (Figure 3g,i). These associations reinforce the crucial function of BAG3 in selective autophagy pathways, as previously suggested by studies of protein quality control [29]. The correlation between pre-cathepsin D and mature-cathepsin D levels likely reflects normal lysosomal protease processing, indicating preserved lysosomal function in patient fibroblasts (Figure 3j).

Particularly interesting was the strong negative correlation between pre-cathepsin D and TFE3 (Figure 3m), which appears to contradict the role of TFE3 in promoting lysosomal biogenesis [31]. This unexpected finding warrants further investigation into potential feedback mechanisms regulating lysosomal function. Our analysis also revealed significant correlations involving endosomal trafficking components. EEA1, a key regulator of early endosomal dynamics, showed positive correlations with PSEN2, RAB7, and TFE3, suggesting coordinated regulation of endosomal-lysosomal trafficking in these cells (Figure 3n–p). TOLLIP exhibited multiple significant correlations with other proteins in our dataset, suggesting it may serve as an important coordinator of autophagy-related protein networks (Figure 3e,i,s). This observation extends beyond TOLLIP’s known function in selective autophagy [33] and points to a potentially broader role in maintaining cellular proteostasis. Given the small sample size (*n* = 18), some scatter plots (e.g., in Figure 3d,k,n,p,r) contain visually prominent observations that can substantially influence Spearman’s correlation coefficients and *p*-values. We regard these correlations as exploratory, hypothesis-generating findings, and we did not exclude any data points as statistical outliers.

### 3.5. Integration of AD- and ALP-Associated Features with Clinical/Demographic Data

**Figure 4:**
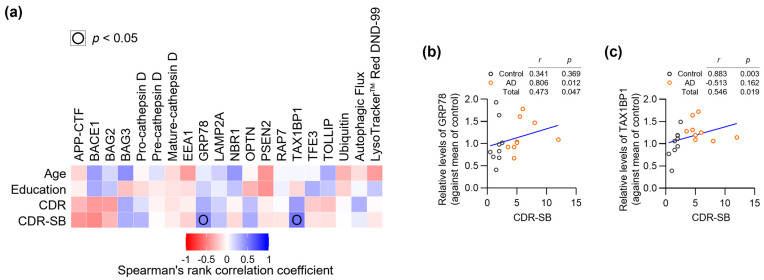
Integration of AD- and ALP-associated features with clinical/demographic data. (**a**) Correlation matrix heatmap showing relationships between all analyzed parameters and clinical/demographic data (*n* = 18). (**b**,**c**) Scatter plots showing correlations between CDR-SB scores and levels of GRP78 (**b**) or TAX1BP1 (**c**). Each point represents an individual patient (Control: *n* = 9, AD: *n* = 9). Spearman’s rank correlation coefficients (*r*) and *p*-values (*p*) are shown for each correlation pair.

To explore the potential clinical relevance of the observed protein alterations in our pilot cohort, we analyzed correlations between AD- and ALP-associated features and clinical/demographic data (Figure 4a and Supplementary Materials Table S1). This exploratory analysis revealed associations with CDR-SB scores, which provide a detailed measure of dementia severity across multiple functional domains [41]. Two proteins showed significant positive correlations with CDR-SB: GRP78 (*r* = 0.473, *p* = 0.047) (Figure 4b) and TAX1BP1 (*r* = 0.546, *p* = 0.019) (Figure 4c). However, when analyzed within individual groups, these correlations showed different patterns: in controls, GRP78 (*r* = 0.341, *p* = 0.369) and TAX1BP1 (*r* = 0.883, *p* = 0.003), and in AD patients, GRP78 (*r* = 0.806, *p* = 0.012) and TAX1BP1 (*r* = −0.513, *p* = 0.162). Given our limited sample size (*n* = 18), these correlation analyses should be interpreted cautiously and require validation in larger cohorts.

The correlation between GRP78 and disease severity is notable, as this ER chaperone protein plays crucial roles in both protein folding and stress-induced autophagy [38]. The observed relationship suggests that cellular stress responses may escalate with advancing clinical symptoms. Similarly, the positive correlation between TAX1BP1 and CDR-SB might reflect increasing activation of selective autophagy mechanisms as disease progresses. These findings suggest that certain stress-response pathways in peripheral tissues may parallel the progression of cognitive decline.

### 3.6. Evaluation of Diagnostic Performance of the Fibroblast Biomarker Models

**Figure 5:**
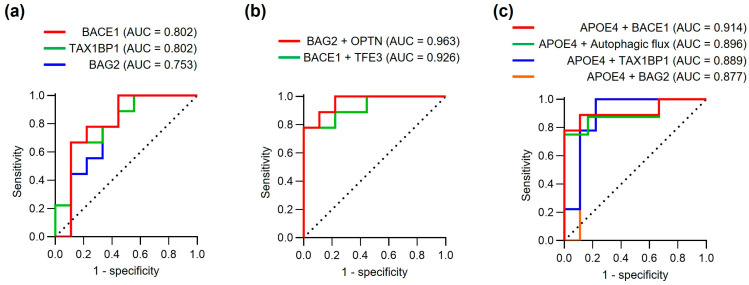
Diagnostic performance of fibroblast biomarker models. (**a**) ROC curves assessing the diagnostic performance of individual proteins (BACE1, TAX1BP1, and BAG2) for discriminating AD patients from controls. (**b**) ROC curves showing protein combinations (BAG2 + OPTN and BACE1 + TFE3). (**c**) ROC curves for combinations of *APOE4* status with individual protein markers or autophagic flux. Area under curve (AUC), sensitivity, specificity and other statistical parameters are detailed in Table 3. Note: Performance estimates may be optimistic due to small sample size and lack of independent validation.

To assess the preliminary diagnostic potential of the observed protein alterations in this small exploratory cohort, we performed receiver operating characteristic (ROC) curve analysis (Figure 5 and Table 3). Among individual proteins, BACE1 demonstrated strong discriminatory power (AUC = 0.802, sensitivity 100%, specificity 55.6%), matching the performance of TAX1BP1 (AUC = 0.802, sensitivity 66.7%, specificity 88.9%). BAG2, while showing lower overall accuracy (AUC = 0.753), achieved notably high sensitivity (100%) at the expense of specificity (55.6%, *p* = 0.038) (Figure 5a).

To explore whether protein combinations might improve diagnostic accuracy, we evaluated various protein combinations using logistic regression analysis (Figure 5b). The combination of BAG2 and OPTN showed promising preliminary performance (AUC = 0.963, sensitivity 77.8%, specificity 100%), surpassing all individual markers in this dataset. Similarly, combining BACE1 and TFE3 achieved excellent discriminatory power (AUC = 0.926, sensitivity 77.8%, specificity 100%). These exploratory results suggest that multi-protein panels may provide more reliable diagnostic performance than single markers, though validation in independent cohorts is essential to confirm these findings. However, these multivariable models were derived from only 18 subjects and have a very low EPV for two predictors, so their ROC estimates are likely to be overly optimistic and prone to overfitting. Accordingly, all multivariable models in this study (e.g., BAG2 + OPTN, BACE1 + TFE3, *APOE4* + BACE1) should be interpreted strictly as exploratory, hypothesis-generating tools rather than definitive diagnostic models.

Given the established role of *APOE4* as a major genetic risk factor for AD [42], we investigated whether incorporating *APOE4* status could further enhance diagnostic accuracy. Indeed, combining *APOE4* with individual protein markers consistently improved performance, with *APOE4* + BACE1 showing particularly strong results (AUC = 0.914, sensitivity 88.9%, specificity 88.9%,). Notably, the combination of *APOE4* with autophagic flux measurements also demonstrated robust diagnostic potential (AUC = 0.896, sensitivity 75.0%, specificity 100%,), suggesting that functional autophagic measures may complement genetic risk assessment.

These preliminary findings suggest that fibroblast-derived protein markers, particularly when used in combination with *APOE4* status, may represent promising candidates for AD diagnostic tools pending validation. The observed performance of protein combinations in this small cohort suggests that capturing multiple aspects of disease-related cellular dysfunction may be important for diagnostic accuracy, though these findings require confirmation in larger, independent datasets to assess their true diagnostic value and generalizability.

## 4. Discussion

This exploratory pilot study investigated autophagy–lysosomal pathway proteins in dermal fibroblasts as potential peripheral biomarkers for Alzheimer’s disease. In a small cohort of 9 AD patients and 9 age-matched controls, we identified disease-associated alterations in several proteins, most notably reduced BACE1 and elevated TAX1BP1 expression. While protein combinations demonstrated promising preliminary diagnostic performance (BAG2 + OPTN: AUC = 0.963), these findings require validation in larger independent cohorts.

Our findings should be considered within the rapidly evolving landscape of AD biomarker research. Recent advances in blood-based biomarkers, particularly plasma p-tau217 and p-tau181, have demonstrated remarkable diagnostic accuracy in large clinical cohorts [6,7,43]. These markers show high sensitivity for detecting AD pathology and are likely to become primary screening tools given their minimal invasiveness and robust validation [44]. However, cellular biomarkers like those derived from dermal fibroblasts may offer complementary information by revealing cell-autonomous dysfunction in protein quality control pathways. Our findings suggest that AD manifests as a systemic disorder affecting cellular proteostasis beyond the CNS [13,14], consistent with emerging evidence that peripheral tissues can reflect neurodegenerative processes [45,46].

These observations should also be interpreted in the context of previous work using comparable methodologies. Prior studies have reported alterations of APP-processing enzymes and ALP components in AD brain tissue and peripheral samples, supporting a role for dysregulated proteostasis in AD pathophysiology [47,48,49,50,51]. In line with these reports, we found disease-associated changes in BACE1, TAX1BP1, BAG2, and OPTN in dermal fibroblasts, suggesting that disruption of protein quality control pathways may extend beyond the central nervous system. At the same time, the absence of large group differences in autophagic flux and lysosomal acidification in our data, compared with some earlier studies, indicates that fibroblast ALP abnormalities may be more subtle or pathway-specific, and underscores the need for larger cohorts and standardized protocols to clarify these discrepancies.

The reduced BACE1 expression we observed in AD patient fibroblasts contrasts with elevated BACE1 levels typically reported in AD brain tissue [27,28], suggesting tissue-specific regulation of APP processing machinery. This peripheral reduction may represent a compensatory response to systemic metabolic alterations in AD [52], though the biological significance remains unclear and warrants further investigation. The positive correlation between APP-CTF and BACE1 levels supports BACE1’s established role in APP processing [28].

The elevated TAX1BP1 expression in AD fibroblasts may be consistent with a compensatory response to protein homeostasis stress, as TAX1BP1 facilitates autophagic clearance of protein aggregates [35,36] although this interpretation remains speculative given the small sample size. Notably, the direction of TAX1BP1 changes observed in peripheral fibroblasts may not mirror CNS-based reports, potentially reflecting tissue-specific regulation; therefore, these findings should be interpreted cautiously as exploratory. Similarly, the consistent directional changes in BAG2 (decreased) and OPTN (increased) across individual AD patients, although not statistically significant in this small cohort, raise the possibility of coordinated alterations in protein quality control networks [29,32], but these patterns require confirmation in larger studies. The preservation of baseline autophagic flux and lysosomal function was unexpected given well-documented autophagy dysfunction in AD neurons [26,40], suggesting that ALP dysfunction in AD may be pathway-specific rather than systemic [51]. However, the absence of statistically significant differences in lysosomal acidification should be interpreted with caution given the limited sample size (*n* = 9 per group). Previous studies have reported disrupted endolysosomal function, including impaired acidification, in AD fibroblast models [50,53]. Therefore, the present findings may reflect insufficient statistical power (i.e., an increased risk of type II error) rather than preserved lysosomal function.

Regarding diagnostic performance, our preliminary ROC analysis demonstrated promising AUC values, particularly for protein combinations and for models integrating *APOE4* status with fibroblast-derived markers. However, critical caveats must be emphasized. First, these performance metrics were derived from the same small cohort (*n* = 18) used for biomarker discovery, without independent validation or cross-validation. This approach is known to produce optimistic performance estimates, especially in small datasets. Second, we did not apply multiple testing corrections given the exploratory nature of this work. Third, Western blot analysis has inherent semi-quantitative limitations for diagnostic applications. Therefore, the reported diagnostic accuracy should be interpreted as preliminary upper bounds requiring substantial downward revision upon independent validation. More clinically applicable quantitative methods such as ELISA or automated immunoassays would be necessary to assess true diagnostic utility.

*APOE4* is also an important context for interpreting our exploratory findings. Large cohort studies have shown that *APOE4* carriers exhibit higher brain amyloid burden and altered plasma Aβ and p-tau profiles, and that accounting for APOE genotype can improve the diagnostic accuracy and risk stratification of fluid biomarkers [6,7,42,43,44]. In our cohort, *APOE4* carrier status was more prevalent in the AD group and systematically enhanced the AUC of fibroblast-based models, with *APOE4* + BACE1 and *APOE4* + autophagic flux showing particularly strong performance. Although the limited sample size precluded stratified analyses by genotype, these exploratory results raise the possibility that *APOE4*-associated mechanisms—such as altered lipid metabolism, endolysosomal trafficking, and proteostasis—may interact with fibroblast ALP alterations. Future studies with larger, genotype-balanced samples will be needed to determine whether fibroblast biomarkers behave differently in *APOE4* carriers versus non-carriers, and whether *APOE4* modifies the relationship between fibroblast markers and clinical progression.

Several important limitations constrain the interpretation of our findings. Most critically, our small sample size (*n* = 9 per group) limits statistical power and increases risks of both false-positive and false- negative findings. Our reliance on Western blot-based quantification, while providing valuable molecular insights, has methodological limitations for diagnostic applications. Additionally, our patient classification was based solely on amyloid PET positivity without comprehensive tau pathology or neurodegeneration biomarkers (A/T/N framework [54]), limiting our ability to correlate peripheral protein changes with specific aspects of AD pathology. Our cohort consisted exclusively of Korean participants from a single center, limiting generalizability to other populations. The cross-sectional design prevents assessment of whether these markers can predict disease progression.

Although the sample size is small and the study is underpowered for definitive conclusions, some meaningful inferences can still be drawn from this exploratory dataset. First, we demonstrate the practical feasibility of using dermal fibroblasts to quantify ALP-related proteins, autophagic flux, and lysosomal acidification in amyloid PET–characterized individuals [50,55,56]. Second, even in this limited cohort, we observed biologically plausible AD-related trends in BACE1, TAX1BP1, BAG2, and OPTN, and a proof-of-concept that combining fibroblast-derived markers with *APOE4* status may improve classification performance. Thus, our data provide hypothesis-generating information that can guide the design and prioritization of future, adequately powered validation studies.

Despite these limitations, our findings contribute to understanding AD as a systemic disorder and suggest that dermal fibroblasts harbor disease-associated molecular signatures. Future validation studies should employ larger multi-center cohorts (target *n* > 100 per group) with diverse populations, independent validation datasets, and standardized quantitative methods. Head-to-head comparison with established blood biomarkers in the same individuals would clarify whether fibroblast markers provide complementary information. Longitudinal studies could assess predictive value for disease progression. If validated and if added clinical value is demonstrated, fibroblast-based approaches could complement blood-based diagnostics in specific research or clinical contexts, though practical considerations including skin biopsy and cell culture requirements limit routine clinical applicability compared to blood-based methods.

## 5. Conclusions

This exploratory pilot study provides preliminary evidence that autophagy–lysosomal pathway proteins in dermal fibroblasts show disease-associated alterations in Alzheimer’s disease. While our findings in a small cohort (*n* = 18) suggest potential diagnostic utility, particularly for the BAG2 + OPTN combination (AUC = 0.963), these results require validation in larger, independent cohorts before clinical translation. Given the limited sample size and lack of independent validation, the reported diagnostic performance estimates are likely optimistic and should be interpreted cautiously. The identified protein signatures may represent peripheral manifestations of AD pathology and could complement established blood-based biomarkers such as plasma p-tau217 and p-tau181 in specific contexts. However, substantial additional work including multi-center validation studies, standardized quantitative methods, and demonstration of added clinical value is required before any clinical application. These preliminary findings provide a foundation for future biomarker development and advance understanding of AD as a systemic disorder affecting cellular proteostasis beyond the central nervous system.

## Figures and Tables

**Table 1:** Demographic and clinical characteristics of subjects.

	Control (*n* = 9)	AD (*n* = 9)	U-Statistic Value	*p*-Value
Sex (male/female)	2/7	1/8	-	0.999 ^a^
Age (years)	69.8 ± 4.5	68.2 ± 5.3	30.50	0.395 ^b^
Education duration (years)	9.4 ± 2.9	6.8 ± 6.4	24.50	0.161 ^b^
CDR	0.5 ± 0.0	1.1 ± 0.4	4.50	<0.001 ^b^
CDR-SB	1.7 ± 0.6	6.0 ± 2.6	0	<0.001 ^b^
*APOE4* (carrier/non-carrier)	2/7	7/2	-	0.057 ^a^

CDR, clinical dementia rating; CDR-SB, CDR sum of boxes. ^a^ *p*-value by Fisher’s exact test. ^b^ *p*-value by Mann–Whitney U test.

**Table 2:** Quantitative analysis of protein levels and autophagic activities of subjects.

Marker	Median (95% CI for Median)		U-Statistic Value	*p*-Value ^a^
	Control	AD		
APP-CTF	1.28 (0.197 to 1.546)	0.84 (0.343 to 1.139)	28	0.430
BACE1	0.90 (0.659 to 1.673)	0.55 (0.279 to 0.786)	16	0.022 *
BAG2	1.04 (0.423 to 1.618)	0.52 (0.194 to 0.870)	20	0.052
BAG3	1.00 (0.427 to 1.367)	1.88 (0.478 to 3.552)	29	0.116
Pro-cathepsin D	0.78 (0.335 to 1.812)	1.19 (0.517 to 1.542)	32	0.772
Pre-cathepsin D	0.77 (0.040 to 2.390)	0.32 (0.130 to 1.040)	36	0.454
Mature-cathepsin D	0.47 (0.340 to 2.650)	0.75 (0.430 to 1.660)	37	0.718
EEA1	0.98 (0.753 to 1.238)	1.21 (0.889 to 1.720)	25	0.271
GRP78	0.83 (0.679 to 1.639)	1.04 (0.923 to 1.606)	27	0.420
LAMP2A	0.62 (0.609 to 1.400)	0.80 (0.702 to 1.638)	30	0.421
NBR1	0.87 (0.781 to 1.490)	0.96 (0.390 to 1.360)	39	0.753
OPTN	1.07 (0.709 to 1.454)	1.65 (0.444 to 2.618)	21	0.078
PSEN2-CTF	1.02 (0.550 to 1.390)	0.96 (0.620 to 2.790)	32	0.248
RAB7	0.97 (0.792 to 1.328)	1.13 (0.560 to 1.622)	34	0.518
TAX1BP1	1.07 (0.772 to 1.191)	1.25 (1.092 to 1.643)	16	0.035 *
TFE3	0.77 (0.490 to 1.690)	0.68 (0.100 to 1.290)	31.5	0.308
TOLLIP	0.84 (0.593 to 1.625)	0.78 (0.528 to 1.264)	31	0.429
Ubiquitin	1.04 (0.675 to 1.208)	1.13 (0.884 to 1.421)	29	0.281
Autophagic flux	1.80 (1.480 to 3.760)	2.20 (1.580 to 4.790)	14	0.312
LysoTracker™ Red DND-99	3792 (2981 to 4646)	3699 (1260 to 5234)	34	0.870

Control, *n* = 9; AD, *n* = 9. Autophagic flux (control, *n* = 6; AD, *n* = 8). LysoTracker™ Red DND-99 (control, *n* = 9; AD, *n* = 8). ^a^ *p*-value by Mann–Whitney U test. * *p* < 0.05.

**Table 3:** Detailed information of ROC curve analysis.

Marker	AUC	Cutoff	Sensitivity (%)	Specificity (%)	*p*-Value ^a^	95% CI for AUC	95% CI for Sensitivity	95% CI for Specificity
BACE1	0.802	0.847	100	55.6	0.013	0.568 to 1.000	0.701 to 1.000	0.267 to 0.811
TAX1BP1	0.802	0.547	66.7	88.9	0.018	0.591 to 1.000	0.354 to 0.879	0.565 to 0.980
BAG2	0.753	0.335	100	55.6	0.038	0.523 to 0.984	0.701 to 1.000	0.267 to 0.811
BAG2 + OPTN	0.963	0.470	77.8	100	0.0004	0.890 to 1.000	0.453 to 0.937	0.701 to 1.000
BACE1 + TFE3	0.926	0.750	77.8	100	0.002	0.806 to 1.000	0.453 to 0.937	0.701 to 1.000
*APOE4* + BACE1	0.914	0.339	88.9	88.9	0.002	0.771 to 1.000	0.565 to 0.980	0.565 to 0.980
*APOE4* + Autophagic flux	0.896	0.311	75.0	100	0.006	0.723 to 1.000	0.409 to 0.929	0.610 to 1.000
*APOE4* + TAX1BP1	0.889	0.319	100	77.8	0.016	0.727 to 1.000	0.701 to 1.000	0.453 to 0.937
*APOE4* + BAG2	0.877	0.288	100	77.8	0.009	0.707 to 1.000	0.701 to 1.000	0.453 to 0.937

Control, *n* = 9; AD, *n* = 9. ^a^ *p*-value by Pearson’s chi-square test. AUC, area under the curve; CI, confidence interval. All multivariable models are exploratory because of the small sample size and low EPV; their AUC, sensitivity, and specificity values would overestimate true diagnostic performance.

## Data Availability

The data supporting the findings of this study are available from the corresponding author upon reasonable request. Due to privacy and ethical restrictions, raw patient-related data cannot be publicly released.

## Supplementary Materials

The following supporting information can be downloaded at: https://www.mdpi.com/article/10.3390/biomedicines14010034/s1, Figure S1: Expression profiles of AD- and ALP-associated proteins in AD patient fibroblasts; Figure S2: Flow cytometry histograms showing LysoTracker™ Red DND-99 fluorescence intensity in control and AD patient fibroblasts; Table S1: Statistical analysis of AD- and ALP-associated features and clinical/demographic data.

## Abbreviations

The following abbreviations are used in this manuscript:

Aβ	Amyloid-beta. A peptide derived from APP cleavage, accumulating in extracellular plaques in AD.
AD	Alzheimer’s disease. A progressive neurodegenerative disorder characterized by cognitive decline and pathological hallmarks such as amyloid plaques and neurofibrillary tangles.
ALP	Autophagy–lysosomal pathway. The cellular degradation system responsible for protein and organelle turnover.
*APOE4*	Apolipoprotein E4. A major genetic risk allele strongly associated with sporadic AD.
APP	Amyloid precursor protein. A membrane protein that is cleaved to generate amyloid-beta peptides.
AUC	Area under the curve. Metric derived from ROC analysis representing model performance.
Autophagic flux	The dynamic process including autophagosome formation, lysosomal fusion, and cargo degradation.
BACE1	Beta-site APP cleaving enzyme 1. Key enzyme initiating amyloidogenic APP processing.
BAG2/BAG3	BCL2-associated athanogene proteins 2 and 3. Co-chaperones regulating protein quality control.
Cathepsin D	Lysosomal protease involved in protein degradation.
CDR/CDR-SB	Clinical Dementia Rating/Clinical Dementia Rating-Sum of Boxes. Clinical scales for assessing dementia severity.
CSF	Cerebrospinal fluid. Biological fluid surrounding the brain and spinal cord, commonly used for biomarker studies.
EEA1	Early endosome antigen 1. Marker for early endosomes.
EOAD	Early-onset Alzheimer’s disease. AD subtype with clinical onset before the age of 65.
EPV	Events per variable. The number of outcome events per predictor variable in a regression model, used to assess overfitting risk and estimate stability in small-sample analyses.
Fibroblast biomarker models	Diagnostic models based on protein expression profiles derived from dermal fibroblasts.
GRP78	Glucose-regulated protein 78. Endoplasmic reticulum stress chaperone.
LAMP2A	Lysosome-associated membrane protein 2A. Receptor for chaperone-mediated autophagy.
LC3	Microtubule-associated protein 1 light chain 3. Marker of autophagosome formation.
LOAD	Late-onset Alzheimer’s disease. Form of AD with onset typically after the age of 65.
Lysosomal acidification	The process of maintaining acidic pH within lysosomes, essential for enzymatic activity.
NBR1	Neighbor of BRCA1 gene 1. Autophagy receptor involved in protein aggregate clearance.
OPTN	Optineurin. Autophagy receptor involved in selective degradation pathways.
PET	Positron emission tomography. Imaging method for assessing amyloid and tau pathology in vivo.
PSEN1/2	Presenilin 1/2. Components of the γ-secretase complex involved in APP processing.
RAB7	RAS-Associated Protein RAB7A. Small GTPase regulating late endosomal trafficking and lysosomal function.
ROC	Receiver operating characteristic. Statistical tool to evaluate diagnostic accuracy.
SIMOA	Single molecule array. An ultrasensitive immunoassay technology for protein detection.
SUVR	Standard uptake value ratio. Quantitative measure used in PET imaging.
TAX1BP1	Tax1-binding protein 1. Autophagy adaptor protein involved in selective degradation.
TFE3	Transcription factor E3. Regulator of lysosomal biogenesis and autophagy genes.
TOLLIP	F. Autophagy-related adaptor protein.
Ubiquitin	Small protein tag that signals for protein degradation.
WMH	White matter hyperintensity. Lesions observed on MRI, associated with cerebrovascular pathology.
