# Supplementary material for: Exploratory Analysis of Autophagy–Lysosomal Pathway Proteins in Dermal Fibroblasts as Potential Peripheral Biomarkers for Alzheimer’s Disease: A Pilot Study
Source: Biomedicines. 2025 Dec 23;14(1):34. doi: 10.3390/biomedicines14010034 (PMC12838323; doi:10.3390/biomedicines14010034)
Supplement: Supplementary file 1 [file biomedicines-14-00034-s001.zip › biomedicines-4029934-supplementary.pdf]

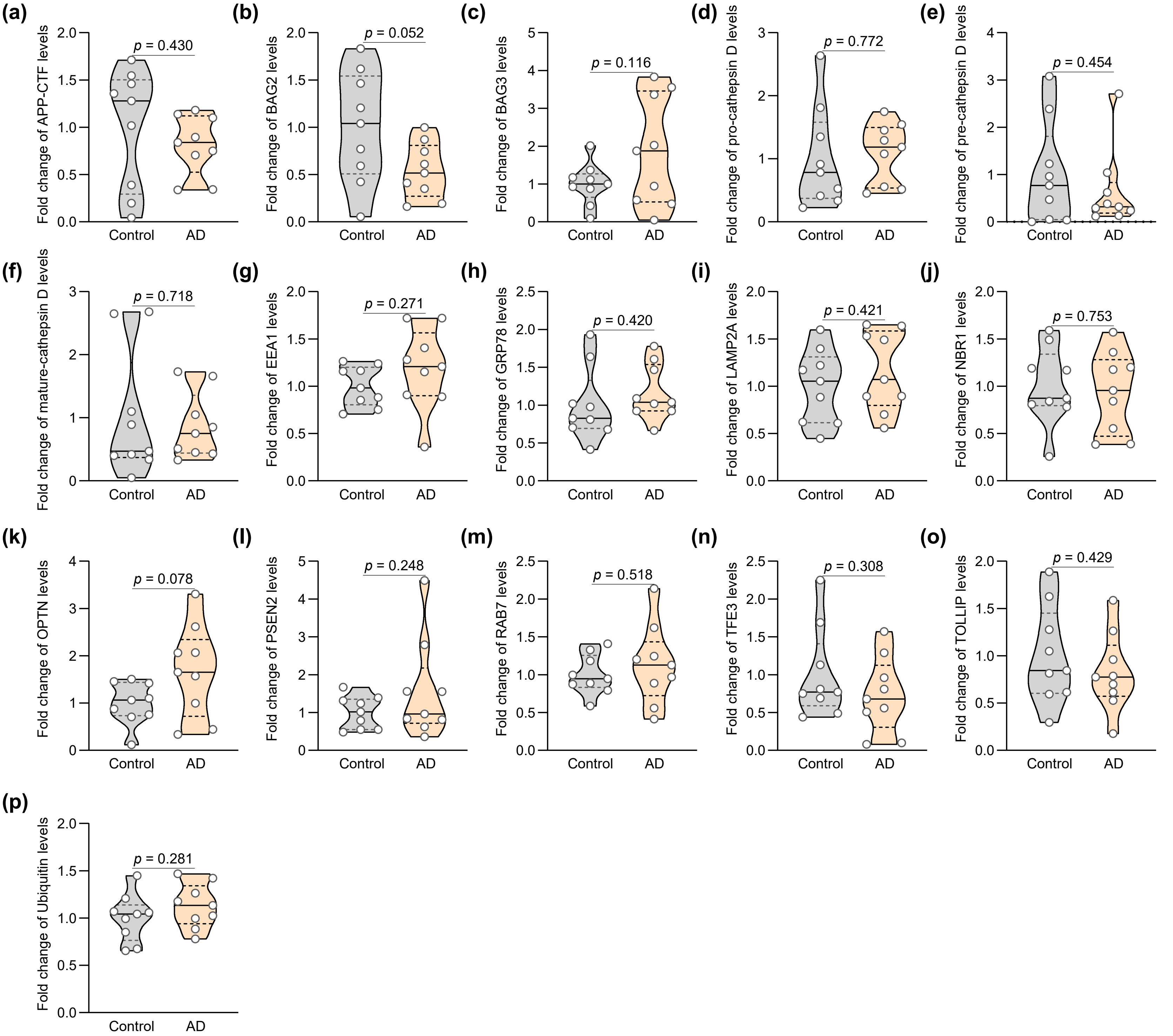

Figure S1. Expression profiles of AD- and ALP-associated proteins in AD patient fibroblasts.

(a-p) Quantification of protein levels of APP-CTF (**a**), BAG2 (**b**), BAG3 (**c**), Pro-cathepsin D (**d**), Pre-cathepsin D (**e**), Mature-cathepsin D (**f**), EEA1 (**g**), GRP78 (**h**), LAMP2A (**i**), NBR1 (**j**), OPTN (**k**), PSEN2 (**l**), RAB7 (**m**), TFE3 (**n**), TOLLIP (**o**), and Ubiquitin (**p**). Data are presented as median ± 95% CI (Control: n=9, AD: n=9). Statistical significance was determined using Mann-Whitney U test ($p < 0.05$).

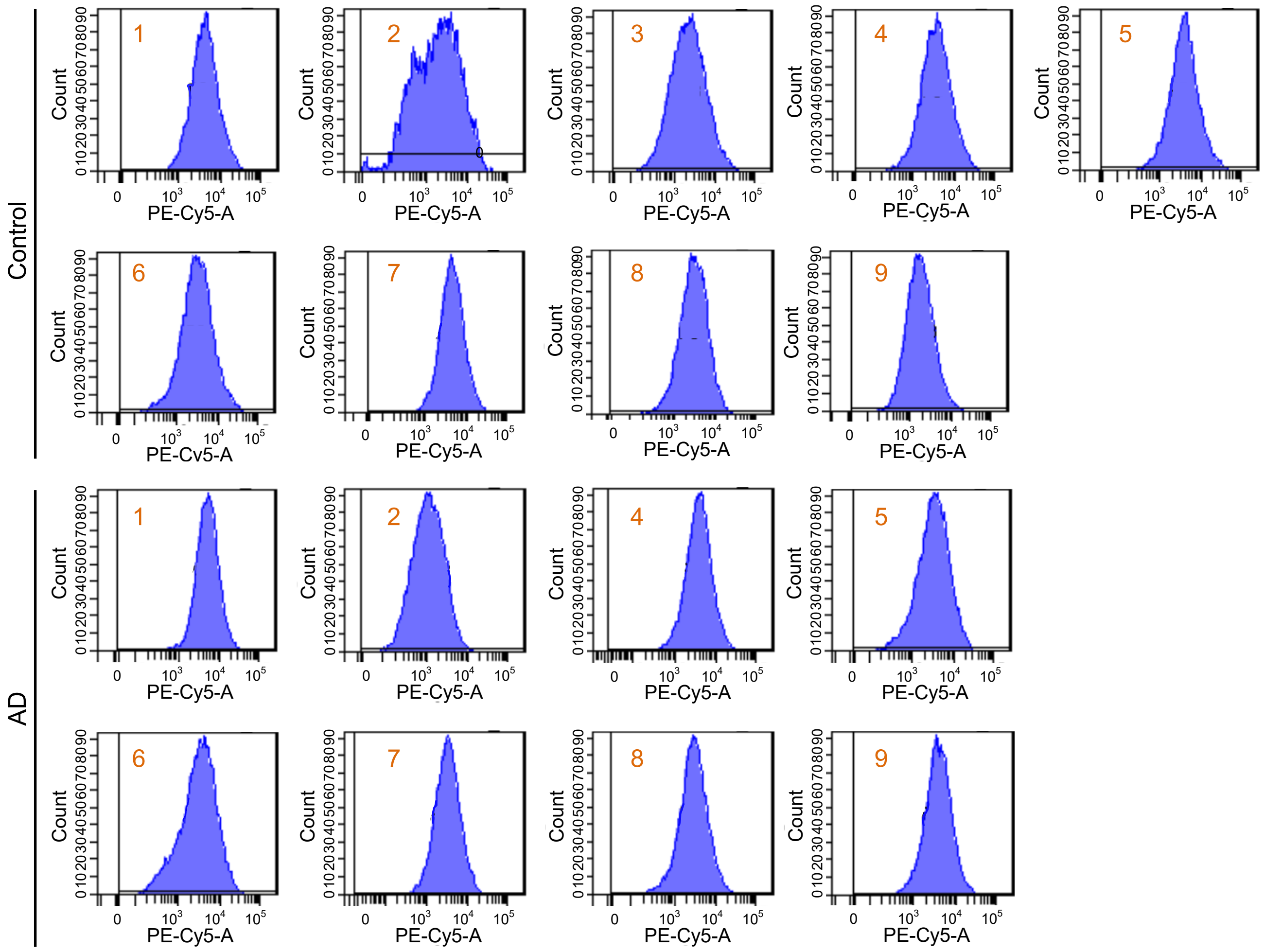

Figure S2. Flow cytometry histograms showing LysoTracker^{TM} Red DND-99 fluorescence intensity in control and AD patient fibroblasts.

Table S1. Statistical analysis of AD- and ALP-associated features and clinical/demographic data

	APP-CTF	BACE1	BAG2	BAG3	Pro-cathepsin D	Pre-cathepsin D	Mature-cathepsin D	EEA1	GRP78	LAMP2A	NBR1	OPTN	PSEN2	RAB7	TAX1BP1	TFE3	TOLLIP	Ubiquitin	LysoTracker™ Red DND-99	Autophagic flux
BACE1	r = 0.475<br>*p = 0.046	-	-	-	-	-	-	-	-	-	-	-	-	-	-	-	-	-	-	-
BAG2	r = 0.012<br>p = 0.961	r = 0.253<br>p = 0.311	-	-	-	-	-	-	-	-	-	-	-	-	-	-	-	-	-	-
BAG3	r = 0.233<br>p = 0.351	r = 0.298<br>p = 0.229	r = 0.135<br>p = 0.593	-	-	-	-	-	-	-	-	-	-	-	-	-	-	-	-	-
Pro-cathepsin D	r = 0.138<br>p = 0.584	r = -0.143<br>p = 0.570	r = 0.075<br>p = 0.766	r = 0.220<br>p = 0.381	-	-	-	-	-	-	-	-	-	-	-	-	-	-	-	-
Pre-cathepsin D	r = 0.396<br>p = 0.104	r = 0.577<br>*p = 0.012	r = -0.139<br>p = 0.581	r = 0.170<br>p = 0.499	r = 0.352<br>p = 0.152	-	-	-	-	-	-	-	-	-	-	-	-	-	-	-
Mature-cathepsin D	r = -0.122<br>p = 0.630	r = -0.203<br>p = 0.418	r = 0.088<br>p = 0.730	r = -0.424<br>p = 0.079	r = 0.581<br>*p = 0.012	r = 0.133<br>p = 0.599	-	-	-	-	-	-	-	-	-	-	-	-	-	-
EEA1	r = -0.307<br>p = 0.216	r = -0.436<br>p = 0.071	r = 0.345<br>p = 0.161	r = -0.308<br>p = 0.214	r = -0.064<br>p = 0.801	r = -0.361<br>p = 0.141	r = 0.340<br>p = 0.168	-	-	-	-	-	-	-	-	-	-	-	-	-
GRP78	r = -0.141<br>p = 0.578	r = -0.018<br>p = 0.095	r = 0.214<br>p = 0.394	r = 0.168<br>p = 0.504	r = 0.416<br>p = 0.086	r = 0.245<br>p = 0.328	r = 0.387<br>p = 0.112	r = -0.009<br>p = 0.972	-	-	-	-	-	-	-	-	-	-	-	-
LAMP2A	r = -0.406<br>p = 0.095	r = -0.310<br>p = 0.211	r = -0.108<br>p = 0.669	r = -0.174<br>p = 0.491	r = -0.247<br>p = 0.324	r = -0.152<br>p = 0.548	r = -0.089<br>p = 0.726	r = 0.258<br>p = 0.301	r = -0.174<br>p = 0.490	-	-	-	-	-	-	-	-	-	-	-
NBR1	r = -0.497<br>*p = 0.036	r = -0.018<br>p = 0.945	r = 0.375<br>p = 0.125	r = -0.143<br>p = 0.573	r = -0.045<br>p = 0.858	r = -0.297<br>p = 0.231	r = 0.311<br>p = 0.209	r = 0.267<br>p = 0.285	r = 0.196<br>p = 0.435	r = 0.013<br>p = 0.958	-	-	-	-	-	-	-	-	-	-
OPTN	r = 0.192<br>p = 0.445	r = -0.067<br>p = 0.791	r = 0.397<br>p = 0.103	r = 0.529<br>*p = 0.024	r = 0.515<br>*p = 0.029	r = -0.024<br>p = 0.926	r = 0.212<br>p = 0.399	r = 0.439<br>p = 0.069	r = 0.281<br>p = 0.259	r = -0.240<br>p = 0.338	r = 0.182<br>p = 0.471	-	-	-	-	-	-	-	-	-
PSEN2	r = 0.074<br>p = 0.769	r = -0.125<br>p = 0.621	r = 0.222<br>p = 0.376	r = 0.045<br>p = 0.858	r = -0.005<br>p = 0.984	r = -0.107<br>p = 0.672	r = 0.170<br>p = 0.499	r = 0.485<br>*p = 0.042	r = -0.112<br>p = 0.658	r = -0.101<br>p = 0.689	r = -0.164<br>p = 0.515	r = 0.469<br>*p = 0.0497	-	-	-	-	-	-	-	-
RAB7	r = -0.020<br>p = 0.937	r = 0.024<br>p = 0.926	r = 0.108<br>p = 0.669	r = -0.279<br>p = 0.263	r = 0.223<br>p = 0.373	r = 0.137<br>p = 0.587	r = 0.119<br>p = 0.639	r = 0.493<br>*p = 0.038	r = -0.016<br>p = 0.951	r = 0.278<br>p = 0.264	r = 0.295<br>p = 0.235	r = 0.180<br>p = 0.473	r = 0.136<br>p = 0.590	-	-	-	-	-	-	-
TAX1BP1	r = -0.247<br>p = 0.323	r = -0.227<br>p = 0.364	r = -0.087<br>p = 0.732	r = 0.216<br>p = 0.390	r = -0.374<br>p = 0.127	r = -0.222<br>p = 0.376	r = 0.245<br>p = 0.328	r = 0.239<br>p = 0.340	r = 0.241<br>p = 0.335	r = -0.043<br>p = 0.864	r = 0.225<br>p = 0.369	r = 0.353<br>p = 0.150	r = -0.072<br>p = 0.775	r = -0.121<br>p = 0.633	-	-	-	-	-	-
TFE3	r = -0.456<br>p = 0.057	r = -0.254<br>p = 0.309	r = 0.391<br>p = 0.108	r = -0.474<br>*p = 0.047	r = -0.374<br>p = 0.127	r = -0.711<br>*p = 0.0009	r = 0.099<br>p = 0.696	r = 0.493<br>*p = 0.038	r = -0.208<br>p = 0.407	r = 0.284<br>p = 0.253	r = 0.655<br>*p = 0.003	r = -0.150<br>p = 0.553	r = -0.118<br>p = 0.640	r = 0.194<br>p = 0.441	r = -0.146<br>p = 0.564	-	-	-	-	-
TOLLIP	r = 0.348<br>p = 0.157	r = 0.556<br>*p = 0.017	r = 0.523<br>*p = 0.025	r = 0.556<br>*p = 0.017	r = 0.422<br>p = 0.081	r = 0.234<br>p = 0.350	r = 0.084<br>p = 0.742	r = -0.038<br>p = 0.880	r = 0.309<br>p = 0.212	r = -0.254<br>p = 0.309	r = 0.018<br>p = 0.945	r = 0.472<br>*p = 0.048	r = -0.009<br>p = 0.971	r = -0.193<br>p = 0.443	r = 0.186<br>p = 0.460	r = -0.131<br>p = 0.604	-	-	-	-
Ubiquitin	r = 0.064<br>p = 0.801	r = -0.044<br>p = 0.861	r = -0.317<br>p = 0.200	r = -0.296<br>p = 0.233	r = 0.001<br>p = 0.997	r = 0.278<br>p = 0.265	r = 0.146<br>p = 0.565	r = 0.380<br>p = 0.120	r = -0.203<br>p = 0.420	r = -0.066<br>p = 0.795	r = -0.086<br>p = 0.735	r = 0.152<br>p = 0.548	r = -0.367<br>p = 0.135	r = 0.676<br>*p = 0.002	r = -0.159<br>p = 0.528	r = -0.225<br>p = 0.369	r = -0.300<br>p = 0.226	-	-	-
LysoTracker™ Red DND-99	r = 0.351<br>p = 0.167	r = 0.253<br>p = 0.327	r = -0.348<br>p = 0.171	r = -0.208<br>p = 0.421	r = -0.277<br>p = 0.281	r = -0.124<br>p = 0.636	r = -0.159<br>p = 0.540	r = -0.213<br>p = 0.410	r = -0.166<br>p = 0.522	r = -0.038<br>p = 0.885	r = -0.357<br>p = 0.159	r = -0.461<br>p = 0.065	r = -0.256<br>p = 0.318	r = -0.173<br>p = 0.504	r = -0.023<br>p = 0.930	r = 0.020<br>p = 0.942	r = -0.051<br>p = 0.846	r = -0.120<br>p = 0.646	-	-
Autophagic flux	r = -0.101<br>p = 0.730	r = -0.195<br>p = 0.505	r = -0.112<br>p = 0.704	r = -0.126<br>p = 0.667	r = -0.539<br>*p = 0.047	r = -0.110<br>p = 0.673	r = -0.402<br>p = 0.155	r = 0.398<br>p = 0.160	r = -0.248<br>p = 0.391	r = 0.513<br>p = 0.063	r = -0.130<br>p = 0.660	r = -0.117<br>p = 0.693	r = 0.033<br>p = 0.916	r = 0.306<br>p = 0.285	r = 0.200<br>p = 0.489	r = 0.231<br>p = 0.427	r = -0.297<br>p = 0.303	r = 0.310<br>p = 0.281	r = -0.016<br>p = 0.964	-
Age	r = -0.170<br>p = 0.501	r = 0.385<br>p = 0.115	r = 0.162<br>p = 0.521	r = 0.444<br>p = 0.065	r = -0.029<br>p = 0.909	r = 0.121<br>p = 0.632	r = -0.150<br>p = 0.552	r = -0.388<br>p = 0.112	r = 0.087<br>p = 0.731	r = 0.189<br>p = 0.453	r = 0.413<br>p = 0.089	r = -0.033<br>p = 0.896	r = -0.420<br>p = 0.083	r = 0.029<br>p = 0.909	r = 0.036<br>p = 0.888	r = 0.031<br>p = 0.902	r = 0.335<br>p = 0.174	r = -0.303<br>p = 0.222	r = -0.392<br>p = 0.120	r = -0.138<br>p = 0.661
Education	r = -0.062<br>p = 0.808	r = 0.340<br>p = 0.168	r = 0.232<br>p = 0.354	r = -0.247<br>p = 0.323	r = -0.149<br>p = 0.554	r = -0.083<br>p = 0.743	r = -0.114<br>p = 0.654	r = -0.133<br>p = 0.599	r = 0.186<br>p = 0.459	r = 0.074<br>p = 0.772	r = 0.235<br>p = 0.348	r = -0.295<br>p = 0.234	r = -0.420<br>p = 0.083	r = 0.057<br>p = 0.823	r = -0.146<br>p = 0.564	r = 0.315<br>p = 0.203	r = 0.222<br>p = 0.376	r = -0.184<br>p = 0.465	r = 0.216<br>p = 0.403	r = 0.029<br>p = 0.923
CDR	r = -0.284<br>p = 0.253	r = -0.402<br>p = 0.098	r = -0.390<br>p = 0.110	r = 0.340<br>p = 0.168	r = 0.132<br>p = 0.603	r = -0.045<br>p = 0.860	r = -0.110<br>p = 0.663	r = -0.070<br>p = 0.783	r = 0.333<br>p = 0.177	r = 0.181<br>p = 0.472	r = -0.039<br>p = 0.877	r = 0.256<br>p = 0.305	r = -0.060<br>p = 0.813	r = 0.010<br>p = 0.969	r = 0.395<br>p = 0.105	r = -0.210<br>p = 0.404	r = -0.244<br>p = 0.329	r = 0.017<br>p = 0.945	r = -0.016<br>p = 0.953	r = 0.310<br>p = 0.280
CDR-SB	r = -0.419<br>p = 0.083	r = -0.467<br>p = 0.050	r = -0.287<br>p = 0.249	r = 0.324<br>p = 0.189	r = 0.294<br>p = 0.236	r = -0.012<br>p = 0.961	r = -0.075<br>p = 0.768	r = -0.184<br>p = 0.466	r = 0.473<br>*p = 0.047	r = 0.245<br>p = 0.328	r = -0.151<br>p = 0.549	r = 0.336<br>p = 0.173	r = -0.116<br>p = 0.647	r = -0.105<br>p = 0.680	r = 0.546<br>*p = 0.019	r = -0.114<br>p = 0.569	r = -0.123<br>p = 0.628	r = -0.003<br>p = 0.990	r = -0.094<br>p = 0.719	r = 0.078<br>p = 0.791
ApoE4 (carrier / non-carrier)	p = 0.883	p = 0.489	p = 0.340	p = 0.297	p = 0.667	p = 0.667	p = 0.387	p = 0.249	p = 0.813	p = 0.423	p = 0.328	p = 0.297	p = 0.450	p = 0.748	p = 0.098	p = 0.589	p = 0.931	p = 0.605	p = 0.370	p = 0.081

r: Spearman's rank correlation coefficient

[a]*p*-value by Spearman’s rank correlation
